# Association of the Naples prognostic score with Parkinson disease risk: a prospective cohort study

**DOI:** 10.3389/fnagi.2026.1809301

**Published:** 2026-04-30

**Authors:** Zhiqiang Xu, Xiaoxiao Wang, Gaiqing Yang, Nan Li

**Affiliations:** 1 Department of Neurology, Zhengzhou Central Hospital Affiliated to Zhengzhou University, Zhengzhou, China; 2 Zhengzhou Key Laboratory of Medical Neurology, Zhengzhou, China; 3 Research Center of Clinical Epidemiology, Peking University Third Hospital, Beijing, China

**Keywords:** inflammation, Naples prognostic score, nutrition, Parkinson disease, UK Biobank

## Abstract

**Background:**

The Naples prognostic score (NPS), derived from routine inflammatory and nutritional biomarkers, reflects systemic immune–metabolic status. Its prospective association with Parkinson disease (PD) risk has not been well established.

**Methods:**

We analyzed 317,035 UK Biobank participants recruited in 2006–2010 who were free of PD and malignancy at baseline and had complete NPS component data. NPS was derived from serum albumin, total cholesterol, neutrophil-to-lymphocyte ratio, and lymphocyte-to-monocyte ratio. Incident PD through 31 December 2022 was identified using a validated algorithm. Associations were examined using Cox proportional hazards models with multivariable adjustment.

**Results:**

Over a median follow-up of 13.94 years, 2,298 participants (0.72%) developed PD. Higher NPS was significantly associated with increased PD risk. After multivariable adjustment, each 1-point increase in NPS corresponded to a 13% higher risk (Model 2 adjusted HR = 1.13, 95% CI: 1.07–1.19, *p* < 0.001). Compared with participants with NPS = 0, those with moderate to high NPS scores (2–4 points) had progressively elevated risks (NPS = 2: HR = 1.16, 95% CI: 1.03–1.31; NPS = 3: HR = 1.53, 95% CI: 1.29–1.82; NPS = 4: HR = 2.08, 95% CI: 1.21–3.58; all *p* < 0.05), showing a clear dose–response relationship (*p* for trend < 0.001).

**Conclusion:**

In this large prospective cohort, higher NPS was independently associated with increased PD risk. These findings support the potential role of integrated inflammatory–nutritional status in PD development and suggest that NPS may serve as a readily available marker for risk stratification.

## Introduction

Parkinson disease (PD) is among the most prevalent and fastest-growing neurological disorders worldwide (GBD 2015 Neurological Disorders Collaborator Group, 2017). Over the past 25 years, its global prevalence has more than doubled, affecting over 8.5 million individuals in 2019 (Kurth and Brinks, 2025), and is projected to reach approximately 25.2 million by 2050 (Su et al., 2025), largely driven by population aging and increased life expectancy (Dorsey et al., 2018). As a chronic and progressive neurodegenerative disorder, PD is characterized by core motor manifestations—such as bradykinesia, rigidity, and resting tremor—as well as a broad spectrum of non-motor symptoms, imposing an escalating clinical, societal, and economic burden on patients, caregivers, and healthcare systems (Lu et al., 2024). Pathologically, PD is characterized by progressive dopaminergic neurodegeneration and remains irreversible, with no curative therapies available, underscoring the need for improved risk stratification and primary prevention strategies (Cornelis et al., 2025). However, most established risk factors for PD, including advanced age, male gender, and genetic risk, are largely non-modifiable, while other environmental contributors—such as exposure to toxicants or air pollution—are difficult to mitigate (Hu et al., 2024; Tanner and Ostrem, 2024). Consequently, identifying modifiable and clinically accessible predictors or biomarkers of PD risk has become a critical research priority.

The Naples prognostic score (NPS) is a composite index derived from routinely available laboratory parameters, integrating serum albumin, total cholesterol, neutrophil-to-lymphocyte ratio (NLR), and lymphocyte-to-monocyte ratio (LMR) to reflect systemic nutritional and inflammatory status. NPS was originally developed to reflect the roles of malnutrition and chronic inflammation in various diseases, including malignancies (Chen et al., 2022), cardiovascular diseases (Aydın et al., 2025), and respiratory disorders (Zhu et al., 2023). In recent years, increasing recognition of the link between peripheral inflammation and central nervous system pathology has led to growing interest in NPS in neurological diseases (Beltran-Velasco and Clemente-Suárez, 2025; Xu et al., 2025). By combining low-cost, noninvasive, and clinically accessible biomarkers, NPS offers a pragmatic approach for risk stratification in routine practice.

The individual components of NPS have well-established biological relevance. Serum albumin and total cholesterol serve as classical indicators of nutritional status, with lower levels reflecting malnutrition (Craig, 1987; Fabricio, 1991), whereas NLR and LMR are well-established inflammatory markers indicative of immune imbalance and systemic inflammation (Fest et al., 2019; Liu et al., 2024). Nevertheless, the association between NPS and the risk of incident PD has not been well characterized in large prospective population-based studies. Consistent with this, epidemiological studies have reported associations between individual components—particularly NLR and LMR—and the risk or severity of PD (Li et al., 2024). In addition, related composite indices, such as the neutrophil percentage-to-albumin ratio (NPAR), have also been linked to PD risk in population-based studies (Ke et al., 2025), supporting the potential utility of integrated inflammatory–nutritional markers.

However, these markers generally capture isolated aspects of systemic status. An integrated index such as NPS may provide a more comprehensive assessment by simultaneously reflecting both inflammatory and nutritional dimensions. To date, the association between NPS and incident PD has not been well characterized in large prospective population-based cohorts. To address this knowledge gap, we leveraged a large prospective cohort of UK Biobank participants to examine the association between NPS and incident PD. This study aimed to provide epidemiological evidence on whether an integrated inflammation- and nutrition-related measure is associated with PD risk.

## Methods

### Study participants

The UK Biobank is a large prospective cohort study that recruited over 500,000 adults aged 37–73 years between 2006 and 2010. Baseline data, including standardized touchscreen questionnaires, physical examinations, and biological samples, were collected according to previously validated protocols (Sudlow et al., 2015), and detailed variable distributions are publicly available via the UK Biobank Data Showcase. All participants provided electronic informed consent, and the study was approved by the relevant research ethics committee(s).

Among 502,235 participants enrolled in the UK Biobank at baseline (2006–2010), we first excluded individuals with prevalent Parkinson disease at baseline (*n* = 932). We further excluded participants who withdrew from the study or were lost to follow-up (*n* = 1,294). Subsequently, participants with missing data on NPS components (*n* = 84,375), polygenic risk score (PRS) (*n* = 5,167), or covariates (*n* = 65,389) were excluded. In addition, individuals with baseline cancer (*n* = 28,043) were excluded because malignancy may substantially influence inflammatory and nutritional biomarkers. After these exclusions, a total of 317,035 participants were included in the final analysis (Supplementary Figure S1). Baseline characteristics of included and excluded participants are presented in Supplementary Table S1 to assess potential selection bias.

### Outcomes

Incident PD was identified using the UK Biobank–recommended algorithm (reported positive predictive value: 91%) (Hughes et al., 2002; Zheng et al., 2023; Geng et al., 2024), integrating baseline self-reported diagnoses and medication data with linked hospital admission and death registry records (Supplementary Table S2). Follow-up accrued from baseline assessment to PD diagnosis, death, withdrawal, or 31 December 2022, whichever occurred first.

### Naples prognostic score

Consistent with prior study (Xu et al., 2025), the NPS was calculated using four laboratory parameters, as detailed in Supplementary Table S3. For component-wise analyses, each NPS component was modeled as a binary indicator (abnormal = 1 vs. normal = 0). According to the number of abnormal parameters, NPS ranged from 0 to 4. In addition, NPS was analyzed as an ordinal variable and further classified into five levels (NPS = 0, 1, 2, 3, and 4) to evaluate dose–response associations. In categorical analyses, NPS = 0 served as the reference group.

### Covariates

According to prior studies (Xu et al., 2025; Belete et al., 2023; Peng et al., 2024; Wang et al., 2024), variables potentially associated with both NPS and PD were included as covariates, including age, gender, education level, Townsend Deprivation Index (TDI) group, lifestyle factors (smoking status, drinking, physical activity, and body mass index), health conditions (hypertension, diabetes, and stroke), and PD polygenic risk score (PRS). Detailed definitions of covariates and information on missing data are provided in Supplementary Tables S4, S5.

### Statistical analysis

Descriptive statistics were used to summarize baseline characteristics. Continuous variables were presented as mean ± standard deviation or median (interquartile range), as appropriate, and categorical variables as counts (percentages).

Cox proportional hazards models were applied to estimate the associations of NPS and genetic risk with incident Parkinson disease (PD), with results reported as hazard ratios (HRs) and 95% confidence intervals (CIs). Three models with progressive adjustment were constructed: the crude model was unadjusted; Model 1 was adjusted for age, gender, and White ethnicity; Model 2 was further adjusted for education, Townsend Deprivation Index (TDI) group, drinking status, smoking status, body mass index categories, physical activity, hypertension, diabetes, stroke, statin use, and PD polygenic risk score.

NPS was analyzed both as a continuous variable (per 1-point increase) and as a categorical variable (0 [reference], 1, 2, 3, and 4). Kaplan–Meier curves were generated to estimate cumulative incidence across NPS groups and compared using the log-rank test. Restricted cubic spline models were used to examine the potential nonlinear dose–response relationship between NPS and PD risk.

Several sensitivity analyses were performed to assess the robustness of the findings. First, participants who developed PD within the first 2 or 5 years of follow-up were excluded to minimize reverse causation. Second, analyses were repeated after including participants with baseline cancer. Third, analyses were stratified by follow-up duration (0–5 years vs. > 5 years). Subgroup analyses were conducted by including interaction terms to evaluate potential effect modification.

The primary analysis was conducted using complete-case data. As sensitivity analyses, multiple imputation was applied for missing covariates, and the main analyses were repeated in the imputed dataset. In addition, inverse probability weighting was used to account for potential selection bias due to exclusion related to missing NPS components.

The proportional hazards assumption was assessed using Schoenfeld residuals.

Subgroup and interaction analyses were considered exploratory; therefore, no formal adjustment for multiple comparisons was applied, and these findings should be interpreted cautiously.

All analyses were performed using R version 4.4.3. A two-sided *p* value <0.05 was considered statistically significant.

## Results

### Baseline characteristics

Baseline characteristics of the 317,035 participants are presented in Table 1. Over a median follow-up of 13.94 years (IQR: 13.23–14.61), 2,298 participants (0.72%) developed Parkinson disease (PD). The mean age of the cohort was 56.01 ± 8.11 years, and 51.73% were female. Polygenic risk scores were categorized into tertiles, each comprising approximately one-third of participants. NPS ranged from 0 to 4, with 33.64% of participants scoring 0, 41.18% scoring 1, 20.83% scoring 2, 4.16% scoring 3, and 0.19% scoring 4. Participants who developed PD were older and more frequently male, and were more likely to have lower educational attainment.

**Table 1:** Baseline characteristics by incident Parkinson disease status.

Characteristics	Total	Incident Parkinson disease		*p* value
		No	Yes	
Participants, *N*	317,035	314,737	2,298	
Age, y, mean ±SD	56.01 ± 8.11	55.96 ± 8.11	62.46 ± 5.61	<0.001
Age group, *n* (%)				<0.001
<65	262,584 (82.82)	261,292 (83.02)	1,292 (56.22)	
≥65	54,451 (17.18)	53,445 (16.98)	1,006 (43.78)	
Female, *n* (%)	163,989 (51.73)	163,179 (51.85)	810 (35.25)	<0.001
White, *n* (%)	289,457 (91.30)	287,316 (91.29)	2,141 (93.17)	<0.01
Education, *n* (%)				<0.001
Low education level	206,329 (65.08)	204,728 (65.05)	1,601 (69.67)	
High education level	110,706 (34.92)	110,009 (34.95)	697 (30.33)	
TDI group, *n* (%)				0.57
Low deprivation	65,798 (20.75)	65,305 (20.75)	493 (21.45)	
Medium deprivation	192,775 (60.81)	191,378 (60.81)	1,397 (60.79)	
High deprivation	58,462 (18.44)	58,054 (18.45)	408 (17.75)	
BMI, kg/m^2^, mean ± SD	27.30 ± 4.68	27.30 ± 4.68	27.74 ± 4.44	<0.001
BMI group, *n* (%)				<0.001
Normal/Underweight	106,677 (33.65)	106,026 (33.69)	651 (28.33)	
Overweight	136,380 (43.02)	135,311 (42.99)	1,069 (46.52)	
Obese	73,978 (23.33)	73,400 (23.32)	578 (25.15)	
Physical activity, *n* (%)				0.12
Not meeting guidelines	117,485 (37.06)	116,597 (37.05)	888 (38.64)	
Meeting guidelines	199,550 (62.94)	198,140 (62.95)	1,410 (61.36)	
Smoking status, *n* (%)				
Never	174,655 (55.09)	173,454 (55.11)	1,201 (52.26)	<0.001
Previous	110,007 (34.70)	109,052 (34.65)	955 (41.56)	
Current	32,373 (10.21)	32,231 (10.24)	142 (6.18)	
Drinking, *n* (%)	294,555 (92.91)	292,507 (92.94)	2,048 (89.12)	<0.001
Hypertension, *n* (%)	81,918 (25.84)	81,060 (25.75)	858 (37.34)	<0.001
Diabetes, *n* (%)	15,184 (4.79)	14,951 (4.75)	233 (10.14)	<0.001
Stroke, *n* (%)	4,225 (1.33)	4,146 (1.32)	79 (3.44)	<0.001
Statin use, *n* (%)	46,585 (14.69)	45,954 (14.60)	631 (27.46)	<0.001
Lymphocyte, 1,000 cells/μL, median (IQR)	1.88 (1.51, 2.29)	1.88 (1.51, 2.29)	1.80 (1.43, 2.20)	<0.001
Monocyte, 1000 cells/μL, median (IQR)	0.45 (0.37, 0.57)	0.45 (0.37, 0.57)	0.48 (0.39, 0.60)	<0.001
Neutrophill, 1000 cells/μL, median (IQR)	4.00 (3.25, 4.92)	4.00 (3.24, 4.92)	4.11 (3.39, 5.07)	<0.001
Albumin, g/L, median (IQR)	45.26 (43.57, 46.98)	45.26 (43.57, 46.98)	44.88 (43.17, 46.67)	<0.001
Cholesterol, mg/dL, median (IQR)	218.37 (189.99, 247.95)	218.45 (190.06, 247.99)	209.92 (177.79, 241.11)	<0.001
LMR, median (IQR)	4.18 (3.25, 5.33)	4.19 (3.25, 5.33)	3.80 (3.00, 4.97)	<0.001
NLR, median (IQR)	2.13 (1.67, 2.75)	2.13 (1.67, 2.75)	2.28 (1.79, 3.00)	<0.001
Genetic risk, *n* (%)				<0.001
Low	105,679 (33.33)	105,137 (33.40)	542 (23.59)	
Intermediate	105,678 (33.33)	104,952 (33.35)	726 (31.59)	
High	105,678 (33.33)	104,648 (33.25)	1,030 (44.82)	
NPS, *n* (%)				<0.001
NPS = 0	106,660 (33.64)	106,093 (33.71)	567 (24.67)	
NPS = 1	130,566 (41.18)	129,676 (41.20)	890 (38.73)	
NPS = 2	66,034 (20.83)	65,411 (20.78)	623 (27.11)	
NPS = 3	13,188 (4.16)	12,983 (4.13)	205 (8.92)	
NPS = 4	587 (0.19)	574 (0.18)	13 (0.57)	
Follow up, y, median (IQR)	13.94 (13.23, 14.61)	13.95 (13.24, 14.61)	10.04 (7.35, 12.00)	<0.001

NPS, Naples prognostic score; IQR, interquartile range; SD, standard deviation; BMI, body mass index; TDI, Townsend Deprivation Index; NLR, neutrophil-to-lymphocyte ratio; LMR, lymphocyte-to-monocyte ratio.

They also had higher BMI and a greater prevalence of hypertension, diabetes, stroke, and statin use. In addition, PD cases were more likely to be former smokers and less likely to be current drinkers. Laboratory measures showed that participants who developed PD had lower lymphocyte counts, albumin, and cholesterol levels, and higher neutrophil and monocyte counts, as well as higher NLR and lower LMR. Furthermore, PD cases were more frequently classified into higher genetic risk categories and had higher NPS scores, indicating a less favorable inflammatory and nutritional profile.

### Associations between NPS and PD risk

**Figure 1:**
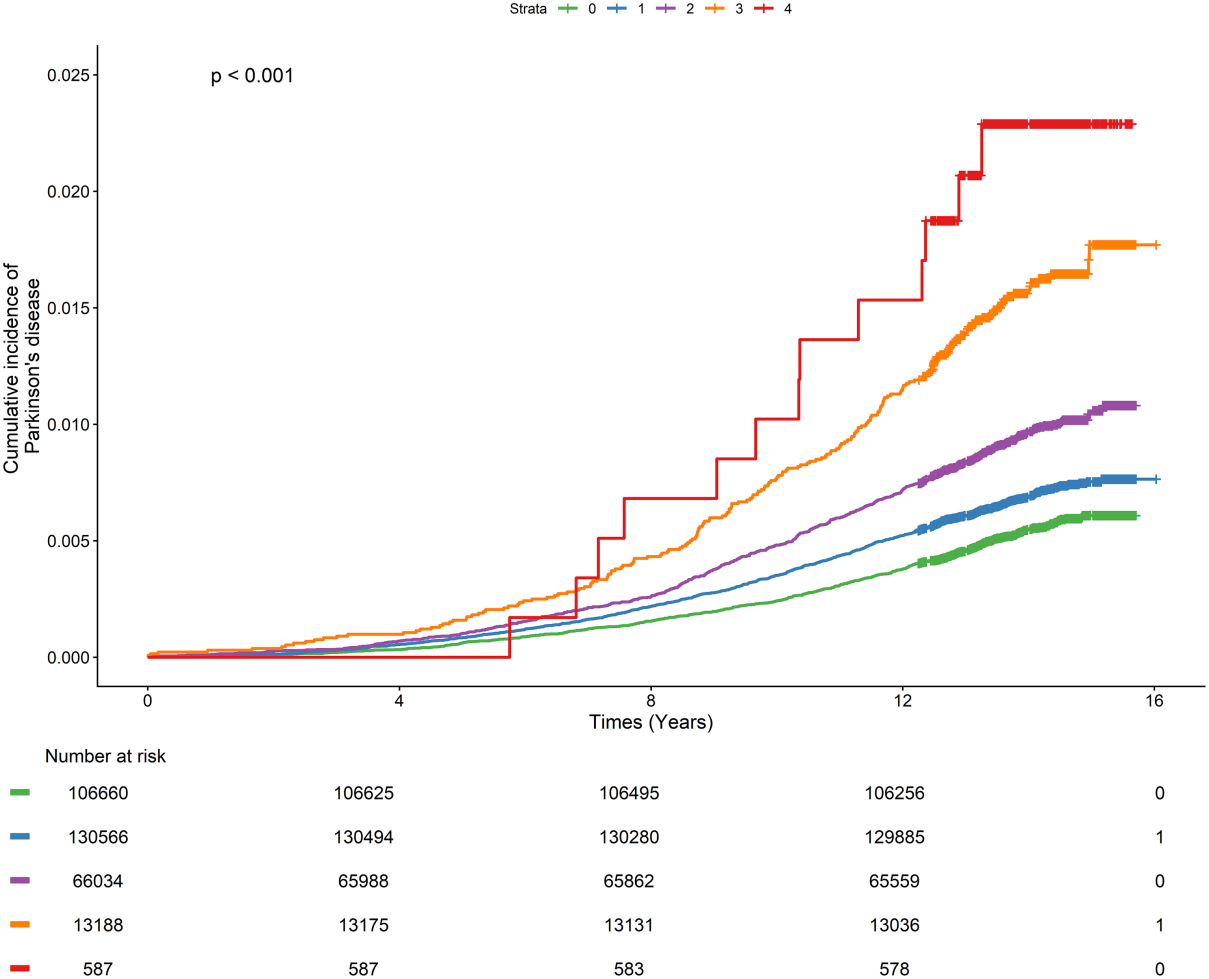
Cumulative incidence of PD by NPS. NPS, Naples prognostic score.

**Figure 2:**
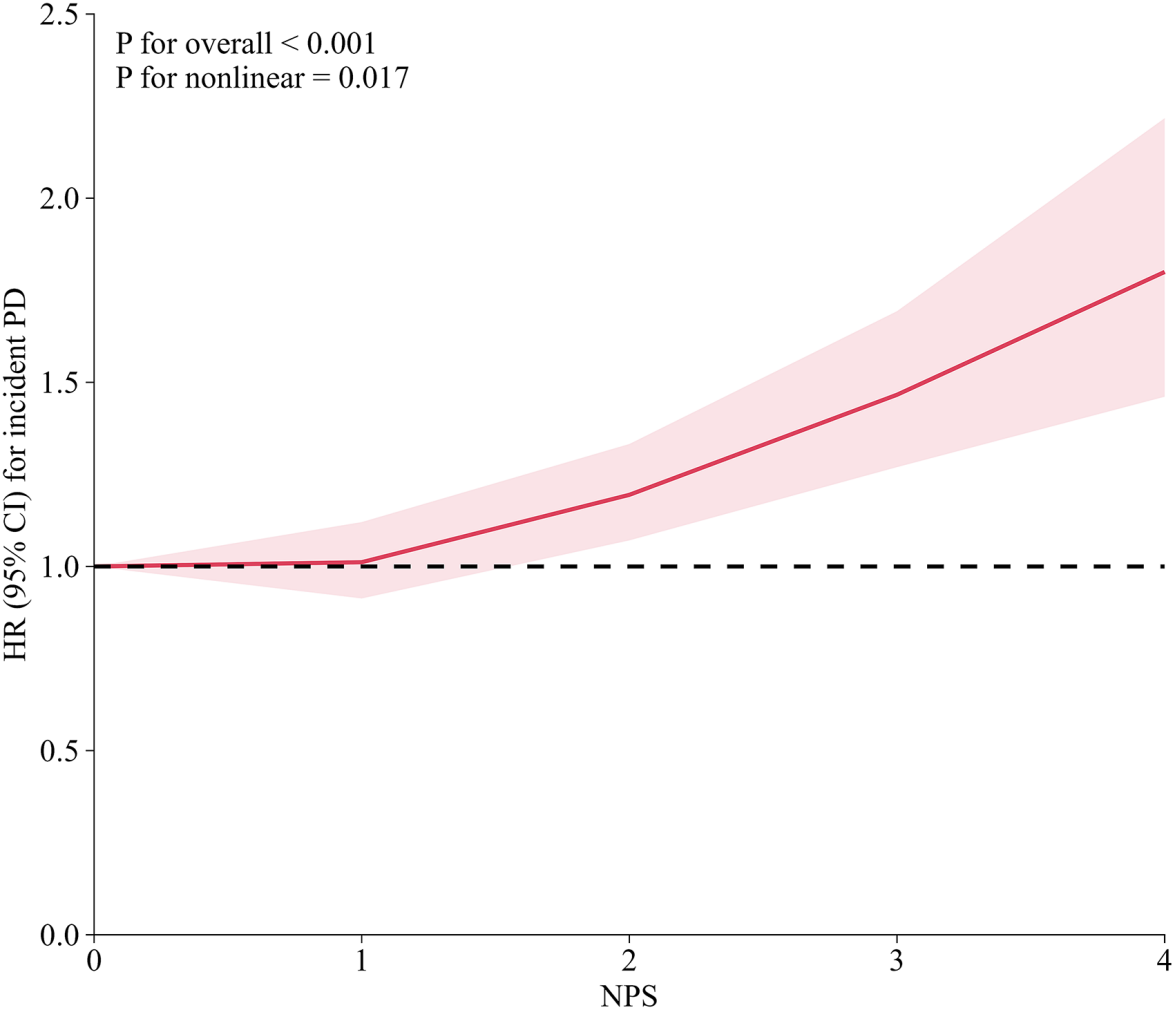
Dose–response association between NPS and incident PD. HR, hazard ratio; CI, confidence interval; PD, Parkinson disease; NPS, Naples prognostic score. Adjusted for age, gender, white ethnicity, education, Townsend deprivation index group, drinking status, smoking status, body mass index, physical activity, hypertension, diabetes, stroke, statin use, and polygenic risk score.

The Kaplan–Meier curves showed that participants with higher NPS had a progressively increased cumulative risk of PD during follow-up, with the highest risk observed in those with NPS = 4 (Figure 1). Restricted cubic spline analysis demonstrated a significant association between NPS and incident PD (*p* for overall <0.001), with evidence of nonlinearity (*p* for nonlinearity = 0.017) (Figure 2). The proportional hazards assumption was assessed using Schoenfeld residuals. The global test did not indicate a significant violation of the proportional hazards assumption (*p* =  0.120), and no evidence of non-proportionality was observed for NPS (*p* = 0.508) (Supplementary Table S6). In Cox proportional hazards models, each 1-point increase in NPS was associated with a 13% higher risk of PD after multivariable adjustment (Model 2 HR = 1.13, 95% CI: 1.07–1.19, *p* < 0.001). Compared with participants with NPS = 0, those with higher NPS levels showed progressively increased risks (NPS = 2: HR = 1.16, 95% CI: 1.03–1.31; NPS = 3: HR = 1.53, 95% CI: 1.29–1.82; NPS = 4: HR = 2.08, 95% CI: 1.21–3.58), with a significant dose–response relationship (*p* for trend <0.001) (Table 2).

**Table 2:** Risk of incident Parkinson disease according to NPS and levels.

Characteristics	Crude model		Model 1		Model 2	
	HR (95% CI)	*p* value	HR (95% CI)	*p* value	HR (95% CI)	*p* value
Per 1-point increase^a^	1.40 (1.33, 1.46)	<0.001	1.17 (1.11, 1.22)	<0.001	1.13 (1.07,1.19)	<0.001
NPS						
0	1 (reference)		1 (reference)		1 (reference)	
1	1.28 (1.15, 1.42)	<0.001	1.05 (0.94, 1.17)	0.40	1.03 (0.93, 1.15)	0.56
2	1.77 (1.58, 1.99)	<0.001	1.22 (1.08, 1.37)	0.001	1.16 (1.03, 1.31)	0.01
3	2.93 (2.49, 3.43)	<0.001	1.71 (1.45, 2.02)	<0.001	1.53 (1.29, 1.82)	<0.001
4	4.31 (2.51, 7.40)	<0.001	2.28 (1.32, 3.92)	0.003	2.08 (1.21, 3.58)	0.01
*p* for trend		<0.001		<0.001		<0.001

Crude model was unadjusted model. Model 1 was adjusted for age, gender, white. Model 2 was adjusted for Model 1 plus education, townsend deprivation index group, drinking status, smoking status, body mass index group, physical activity, hypertension, diabetes, stroke, statin use, and polygenic risk score. ^a^Per 1-point increment in the NPS (0–4 points). HR, hazards ratio; CI, confidence interval; NPS, Naples prognostic score.

When mutually adjusting for the NPS components and covariates, an abnormal cholesterol component (i.e., low total cholesterol score) was independently associated with higher PD risk (HR, 1.21; 95% CI, 1.08–1.35), as were the albumin component (HR, 1.35; 95% CI, 1.07–1.70) and the NLR component (HR, 1.22; 95% CI, 1.11–1.34) (Supplementary Table S7). Regarding genetic risk, participants with intermediate or high polygenic risk had 34 and 91% higher PD risk, respectively, compared with those at low genetic risk (*p* for trend <0.001) (Supplementary Table S8).

### Sensitivity and subgroup analyses

Several sensitivity analyses were conducted to evaluate the robustness of the main findings. The association between higher NPS and increased risk of PD remained consistent across all analyses. After applying inverse probability weighting to account for selection bias related to missing NPS components, the results were materially unchanged (Supplementary Table S9). Analyses based on multiple imputation for missing covariates yielded comparable estimates (Supplementary Table S10). To address potential reverse causation, we repeated the analyses after excluding PD cases occurring within the first 2 and 5 years of follow-up. The findings remained consistent, with similar dose–response relationships observed (Supplementary Tables S11, S12).

Including participants with baseline cancer produced similar findings (Supplementary Table S13), suggesting that exclusion of these individuals did not bias the results. When stratified by follow-up duration, the association between NPS and PD risk was more evident among participants with longer follow-up (>5 years), whereas estimates in the early follow-up period were less precise due to a limited number of events (Supplementary Table S14).

**Figure 3:**
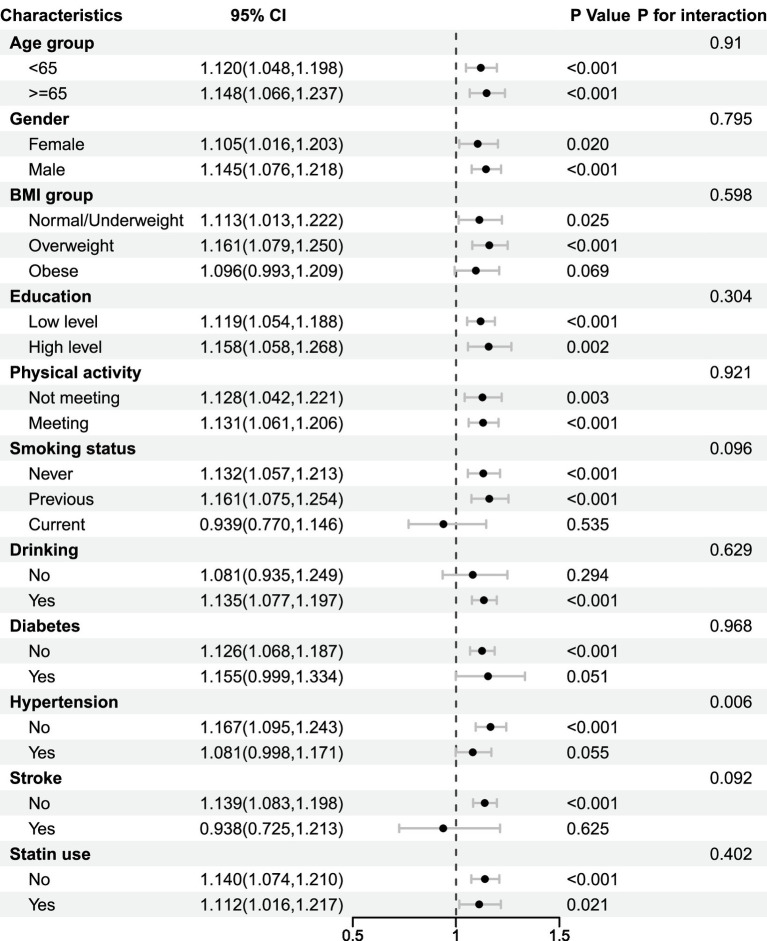
Associations between NPS and incident PD across subgroups. HR, hazard ratio; CI, confidence interval; BMI, body mass index, PRS, polygenic risk score, PD, Parkinson disease. Adjusted for age, gender, White ethnicity, education, townsend deprivation index group, drinking status, smoking status, body mass index, physical activity, hypertension, diabetes, stroke, statin use, and PD polygenic risk score.

In subgroup analyses, evidence of effect modification was observed by hypertension status (*p* for interaction = 0.006), with a stronger association among participants without hypertension. No statistically significant interactions were observed for other subgroups (Figure 3). As these analyses were exploratory and not adjusted for multiple comparisons, the results should be interpreted cautiously.

Overall, the findings were consistent across multiple sensitivity and subgroup analyses.

## Discussion

In this large prospective cohort study of 317,035 participants from the UK Biobank, followed for a median of 13.94 years, higher NPS was associated with a higher risk of incident PD. The association showed a graded pattern across NPS levels and remained after adjustment for sociodemographic factors, lifestyle characteristics, comorbidities, and polygenic risk. These findings suggest that NPS, as an integrated inflammation- and nutrition-related measure, may be associated with PD risk in population-based settings.

Emerging evidence suggests that PD is not solely a disorder of dopaminergic neuronal loss but also involves neuroimmune dysregulation and chronic systemic inflammation (Moquin-Beaudry et al., 2025; Verma et al., 2025). Peripheral immune activity may be linked to central nervous system processes through pathways such as cytokine signaling, blood–brain barrier alterations, and glial activation, which have been associated with oxidative stress and neuronal injury (Kim et al., 2018). Although prior studies have linked individual inflammatory biomarkers to PD risk or severity (Grillo et al., 2023), most have focused on single parameters and cross-sectional designs (Li et al., 2024; Zhang et al., 2024). In contrast, composite indices that integrate inflammatory and nutritional domains may reflect the overall burden of metabolic stress, immune imbalance, and reduced physiological reserve that characterize the prodromal phase of neurodegeneration.

At a biological level, the components of the NPS are related to interconnected metabolic and immune processes that have been implicated in neurodegeneration (Yacoubian et al., 2023). Elevated NLR and altered LMR may indicate a relative predominance of innate immune activity and dysregulated adaptive responses, which have been associated with chronic low-grade inflammation (Li et al., 2024; Muñoz-Delgado et al., 2021). Serum albumin and cholesterol may reflect aspects of systemic metabolic status (Cabrerizo et al., 2015; García-Sanz et al., 2021). Albumin has antioxidant and transport functions (Ke et al., 2025; Liu et al., 2025), whereas cholesterol plays an important role in membrane structure, synaptic organization, and dopaminergic signaling. Lower levels of these factors may be indicative of impaired redox balance and reduced neuronal support, rather than isolated nutritional abnormalities (Tsui-Pierchala et al., 2002; Wang et al., 2017).

Taken together, these findings suggest that NPS reflects an integrated systemic state shaped by nutritional, metabolic, and inflammatory processes. Rather than indicating a PD-specific mechanism, NPS is more likely a nonspecific marker of overall health status. A higher NPS may therefore capture the cumulative burden of physiological dysregulation, including frailty, comorbidities, and subclinical disease, all of which have been linked to increased vulnerability to neurodegeneration. Although individual components of NPS have been associated with neurodegenerative diseases, our results do not support a direct mechanistic link with PD. Instead, the observed association is more likely to reflect broader systemic dysregulation rather than a PD-specific pathogenic pathway or causal relationships. Further mechanistic and experimental studies are needed to clarify the biological relevance of NPS in PD.

### Strengths and limitations

This study has several strengths. Given the limited prospective evidence on the association between a composite inflammatory–nutritional score, NPS, and the risk of incident PD, our analysis based on the large UK Biobank cohort adds longitudinal evidence to the existing literature.

The consistency of our findings is supported by the prospective study design, large sample size, long follow-up duration, and comprehensive adjustment for a wide range of potential confounders, including sociodemographic characteristics, lifestyle factors, comorbidities, and polygenic risk scores. In addition, multiple sensitivity and subgroup analyses, including stratification by genetic risk, were consistent with the main findings, supporting the overall stability of the results.

Several limitations should be acknowledged. First, the relatively low participation rate of the UK Biobank and the predominance of individuals of European ancestry may limit the generalizability of our findings to other populations. Second, although extensive covariate adjustment was performed, residual or unmeasured confounding—particularly from environmental exposures and medication use (e.g., nonsteroidal anti-inflammatory drugs (NSAIDs) and other medications that may influence NPS components)—cannot be fully excluded. In addition, exclusion of participants with missing NPS components or covariates may have introduced selection bias, although inverse probability weighting analyses yielded similar results. Third, reverse causation remains a possibility. Higher NPS levels may reflect subclinical or prodromal PD–related inflammatory and metabolic changes, or declining physiological reserve, rather than etiologic processes, particularly given the long preclinical phase of PD and the reliance on a single baseline measurement of NPS. Fourth, NPS was assessed only at baseline, precluding evaluation of longitudinal changes in inflammatory and nutritional status over time. Time-varying analyses were not performed due to limited availability of repeated measurements in the full cohort. Fifth, the highest NPS category included a relatively small number of participants and events, and the corresponding estimates should be interpreted with caution. Finally, although NPS is derived from routinely available laboratory measures, it is not disease-specific and may reflect broader aspects of systemic health rather than PD-specific mechanisms. Further validation of these findings in more diverse populations, ideally incorporating repeated biomarker assessments, is warranted.

## Conclusion

In summary, higher NPS was independently associated with an increased risk of incident PD in this large prospective cohort. As an integrated measure of inflammatory and nutritional status, NPS may reflect broader systemic conditions associated with PD risk. These findings suggest that NPS may help identify individuals at increased risk of PD in population-based settings, although further studies are needed to clarify its clinical utility.

## Data Availability

Publicly available datasets were analyzed in this study. This data can be found here: This research was conducted using the UK Biobank Resource (application no. 106707). The UK Biobank data are available through application to the UK Biobank (https://www.ukbiobank.ac.uk/), subject to approval and data access policies.
