# Supplementary figures and images for: Association of the Naples prognostic score with Parkinson disease risk: a prospective cohort study
Source: Front Aging Neurosci. 2026 Apr 30;18:1809301. doi: 10.3389/fnagi.2026.1809301 (PMC13171577; doi:10.3389/fnagi.2026.1809301)

Figure S1. Flowchart for the selection of the study population

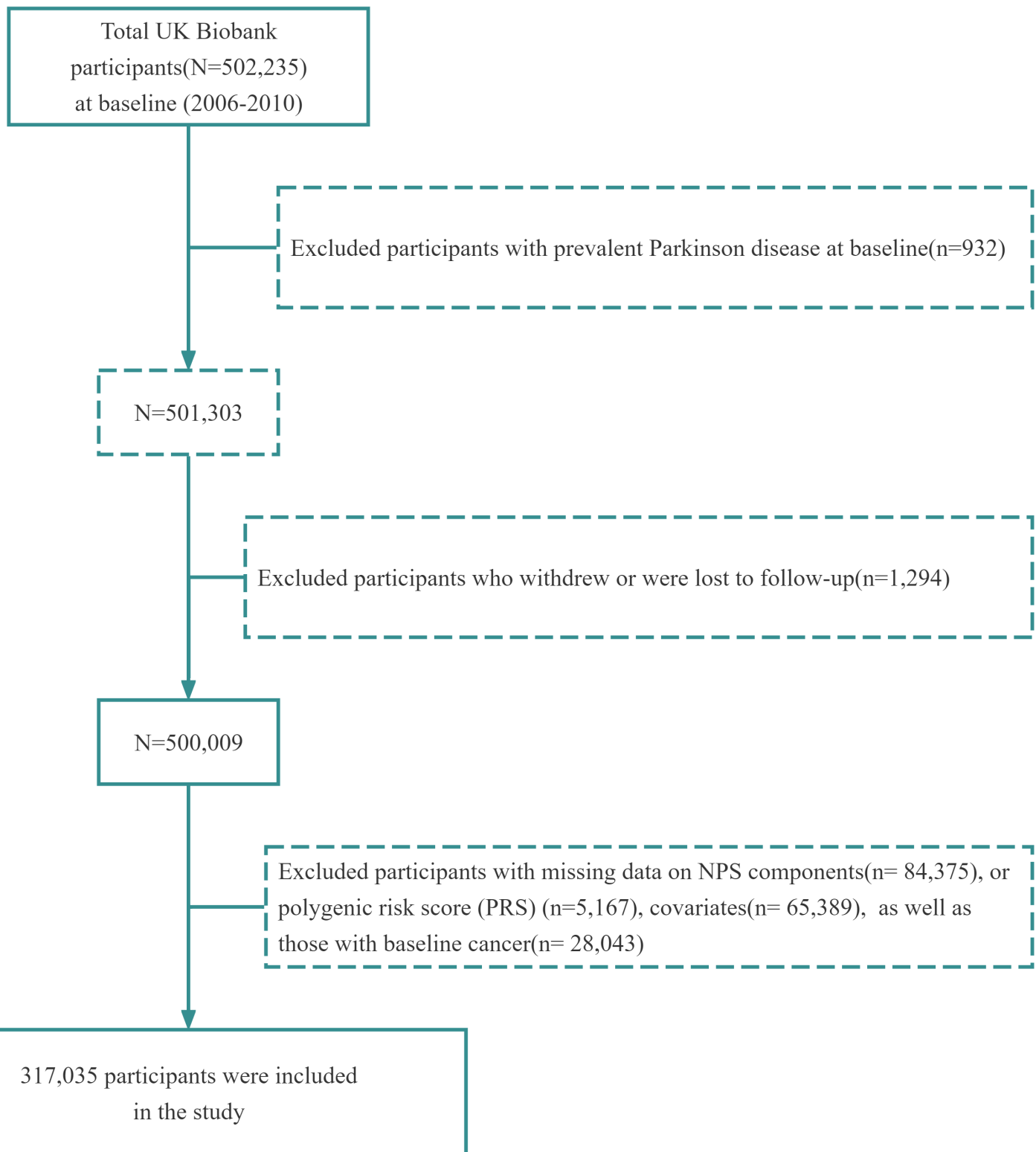

Supplement: Supplementary file 1 [file Data_Sheet_1.pdf]
