# Supplementary material for: Association of the Naples prognostic score with Parkinson disease risk: a prospective cohort study
Source: Front Aging Neurosci. 2026 Apr 30;18:1809301. doi: 10.3389/fnagi.2026.1809301 (PMC13171577; doi:10.3389/fnagi.2026.1809301)
Supplement: Supplementary file 2 [file Supplementary_File_1.docx]

**Table S1.** Comparison of Baseline Characteristics Between Included and Excluded Participants in the UK Biobank

| **Characteristics** | **Total** | **Excluded** | **Included** | ***p* Value** |
| --- | --- | --- | --- | --- |
| **Participants, N** | 502,235 | 185,200 | 317,035 |  |
| **Age, y, mean** ±**SD** | 56.53 ± 8.09 | 57.43 ± 7.99 | 56.01 ± 8.11 | <0.001 |
| **Age group, n (%)** |  |  |  | <0.001 |
| <65 | 406,250(80.89) | 143,666(77.57) | 262,584(82.82) |  |
| ≥65 | 95,985(19.11) | 41,534(22.43) | 54,451(17.18) |  |
| **Female, n (%)** | 273,222(54.40) | 109,233(58.98) | 163,989(51.73) | <0.001 |
| **White, n (%)** | 454,025(90.90) | 164,568(90.21) | 289,457(91.30) | <0.01 |
| **Education, n (%)** |  |  |  | <0.001 |
| Low education level | 331,070(67.28) | 124,741(71.25) | 206,329(65.08) |  |
| High education level | 161,037(32.72) | 50,331(28.75) | 110,706(34.92) |  |
| **TDI group, n (%)** |  |  |  | <0.001 |
| Low deprivation | 100,086(19.95) | 34,288(18.58) | 65,798(20.75) |  |
| Medium deprivation | 301,107(60.03) | 108,332(58.69) | 192,775(60.81) |  |
| High deprivation | 100,416(20.02) | 41,954(22.73) | 58,462(18.44) |  |
| **BMI, kg/m2, mean** ±**SD** | 27.43 ± 4.80 | 27.66 ± 5.00 | 27.30 ± 4.68 | <0.001 |
| **BMI group, n (%)** |  |  |  | <0.001 |
| Normal/Underweight | 164,929(33.04) | 58,252(31.99) | 106,677(33.65) |  |
| Overweight | 212,006(42.48) | 75,626(41.53) | 136,380(43.02) |  |
| Obese | 122,194(24.48) | 48,216(26.48) | 73,978(23.33) |  |
| **Physical activity, n (%)** |  |  |  | <0.001 |
| Not meeting guidelines | 159,750(37.34) | 42,265(38.14) | 117,485(37.06) |  |
| Meeting guidelines | 268,101(62.66) | 68,551(61.86) | 199,550(62.94) |  |
| **Smoking status, n (%)** |  |  |  |  |
| Never | 273,386(54.76) | 98,731(54.17) | 174,655(55.09) | <0.001 |
| Previous | 172,955(34.64) | 62,948(34.54) | 110,007(34.70) |  |
| Current | 52,944(10.60) | 20,571(11.29) | 32,373(10.21) |  |
| **Drinking, n (%)** | 460,124(91.92) | 165,569(90.21) | 294,555(92.91) | <0.001 |
| **Hypertension, n (%)** | 135,707(27.14) | 53,789(29.40) | 81,918(25.84) | <0.001 |
| **Diabetes, n (%)** | 26,390( 5.28) | 11,206( 6.14) | 15,184( 4.79) | <0.001 |
| **Stroke, n (%)** | 7,666( 1.53) | 3,441( 1.88) | 4,225( 1.33) | <0.001 |
| **Statin use, n (%)** | 78,006(15.53) | 31,421(16.97) | 46,585(14.69) | <0.001 |

**Abbreviations:** SD = standard deviation; BMI=body mass index; TDI=Townsend Deprivation Index.

**Table S2.** Ascertainment of Parkinson disease in the UK Biobank study

| **ICD-9** | **ICD-10** | **Self-reported**  **fields** |
| --- | --- | --- |
| 332.0,  332.1,  333.0 | G20, G21, G21.0, G21.1, G21.2, G21.3, G21.4, G21.8, G21.9, G22, G23.0, G23.1, G23.2, G23.3, G23.8, G23.9, G25.9, G26, G90.3 | 20002(1262) |

**Variable definitions were constructed using International Classification of Diseases, Ninth and Tenth Revision (ICD-9 and ICD-10) codes, together with self-reported data fields. Choice-, disease-, or procedure-specific codes are presented in parentheses.**
****Abbreviations:**** ICD, International Classification of Diseases.

**Table S3.** Components and scoring criteria of the Naples Prognostic Score (NPS) in the UK Biobank

| **Domain** | **Component** | **Criteria for Score = 1** | **Criteria for Score = 0** | **UK Biobank Field ID(s)** |
| --- | --- | --- | --- | --- |
| Nutritional status | Serum albumin | < 40 g/L | ≥ 40 g/L | 30600 |
|  | Total cholesterol | ≤ 180 mg/dL | > 180 mg/dL | 30690 |
| Systemic inflammation | NLR | > 2.96 | ≤ 2.96 | 30140 (neutrophil count),  30120 (lymphocyte count) |
|  | LMR | ≤ 4.44 | > 4.44 | 30120 (lymphocyte count),  30130 (monocyte count) |

### ****Note:**** The Naples Prognostic Score (NPS) was calculated as the sum of four dichotomized biomarkers reflecting nutritional status, lipid metabolism, systemic inflammation, and immune balance. Each component was assigned a value of 1 if meeting the unfavorable threshold and 0 otherwise. Higher NPS values indicate poorer nutritional and inflammatory profiles. All biomarker data were obtained from baseline assessments in the UK Biobank. The total NPS ranged from 0 to 4.

Abbreviations: NLR, Neutrophil-to-lymphocyte ratio; LMR,Lymphocyte-to-monocyte ratio.

**Table S4.** Measures of covariates at baseline in the UK Biobank

| **Variable** | **Field ID** | **Question or description** | **Categories from raw data** | **Categories for the current study** |
| --- | --- | --- | --- | --- |
| Education | 6138 | Qualifications | College or University degree;  A levels/AS levels or equivalent;  O levels/GCSEs or equivalent;  CSEs or equivalent; NVQ or HND or HNC or equivalent; Other professional qualifications eg: nursing, teaching. | High level = College or university degree;  Low level = Below college or university degree. |
| Townsend deprivation index gruop | 22189 | Townsend deprivation index at  recruitment | Continuous. | Based on the distribution of TDI in the UK Biobank baseline population, participants were categorized into tertiles:Low deprivation: TDI < –3.93; Medium deprivation: –3.93 ≤ TDI < 1.35; High deprivation: TDI ≥ 1.35. |
| Smoking status | 20116 | The current/past smoking status of the participant | Never;  Past;  Current. | Never = 0;  Previous = 1;  Current = 2. |
| Drinking | 20117 | Alcohol drinker status | Never;  Previous;  Current. | No=0 or 1;  Yes=2. |
| Physical activity | 884,894,  904,914 | Number of days/week of  moderate physical activity 10+ minutes;  Duration of moderate activity;  Number of days/week of  vigorous physical activity 10+ minutes;  Duration of vigorous activity. | Continuous, minutes/day;  days/week. | 1 = < 150 minutes moderate activity per week OR < 75 minutes vigorous activity per week;  0 = ≥150 minutes moderate activity per week OR ≥ 75 minutes vigorous activity per week OR equivalent combination. |
| Body mass index | 21001 | Body mass index (BMI) | kg/m^2^. | Continuous. |
| Hypertension | 6150 | Vascular/heart problems diagnosed by doctor | High blood pressure. | No=1 , 2,3,-7;  Yes=4. |
| Diabetes | 2443 | Diabetes diagnosed by doctor | No;  Yes. | No=0;  Yes=1. |
| Stroke | 6150 | Vascular/heart problems diagnosed by doctor | Stroke. | No=1 , 2,3,-7;  Yes=3. |
| Statin use | 20003 | Treatment/medication code | No;  Yes. | atorvastatin=1141146234  simvastatin=1140861958  fluvastatin=1140888594  pravastatin=1140888648  rosuvastatin=1141192410 |

**Table S5.** Summary of missing data for covariates

| **Covariates** | **Missing (n)** | **Missing (%)** | **Total samples** |
| --- | --- | --- | --- |
| **Age** | 0 | 0 | 410,467 |
| **Gender** | 0 | 0 | 410,467 |
| **Race** | 1,924 | 0.47 | 410,467 |
| **Education** | 4,896 | 1.19 | 410,467 |
| **Townsend deprivation index** | 509 | 0.12 | 410,467 |
| **Body mass index** | 1,613 | 0.39 | 410,467 |
| **Physical activity** | 57,930 | 14.11 | 410,467 |
| **Smoking status** | 2,060 | 0.50 | 410,467 |
| **Drinking status** | 1,014 | 0.25 | 410,467 |
| **Hypertension** | 1,445 | 0.35 | 410,467 |
| **Diabetes** | 1,773 | 0.43 | 410,467 |
| **Stroke** | 1,445 | 0.35 | 410,467 |
| **Statin use** | 0 | 0 | 410,467 |

**Table S6.** Test of Proportional Hazards Assumption Using Schoenfeld Residuals

| **Variable** | **χ²** | **df** | **P value** |
| --- | --- | --- | --- |
| NPS | 0.44 | 1 | 0.508 |
| Age | 5.03 | 1 | 0.025 |
| Gender | 0.01 | 1 | 0.932 |
| White ethnicity | 1.93 | 1 | 0.165 |
| Education | 0.06 | 1 | 0.809 |
| BMI | 1.12 | 1 | 0.291 |
| TDI | 6.64 | 2 | 0.036 |
| Physical activity | 0.56 | 1 | 0.452 |
| Smoking | 0.42 | 2 | 0.809 |
| Drinking | 3.42 | 1 | 0.064 |
| Hypertension | 0.10 | 1 | 0.755 |
| Diabetes | 0.54 | 1 | 0.462 |
| Stroke | 1.18 | 1 | 0.278 |
| Statin use | 1.69 | 1 | 0.193 |
| PD polygenic risk score | <0.001 | 1 | 0.998 |
| Global test | 23.98 | 17 | 0.120 |

The proportional hazards assumption was evaluated using Schoenfeld residuals. A P value greater than 0.05 was considered to indicate no violation of the assumption. Although a few covariates showed statistically significant results, the global test was not significant (P = 0.12), suggesting no evidence of violation at the model level.

**Abbreviations:** df = degrees of freedom; NPS = Naples Prognostic Score; BMI=body mass index; TDI=Townsend Deprivation Index; PD, Parkinson Disease.

**Table S7.** Associations of Individual Components of NPS With Incident Parkinson Disease

| **NPS components^a^** |  | **Cases** | **person-y** |  | **Crude model** | |  | **Model 2** | |
| --- | --- | --- | --- | --- | --- | --- | --- | --- | --- |
|  | **Exposed** |  |  |  | HR(95% CI) | *p* Value |  | HR(95% CI) | *p* Value |
| **Cholesterol score** | 56,636 | 616 | 786,171 |  | 1.69(1.54,1.86) | <0.001 |  | 1.21(1.08,1.35) | <0.001 |
| **Albumin score** | 6,356 | 74 | 88,623 |  | 1.63(1.29,2.05) | <0.001 |  | 1.35(1.07,1.70) | 0.010 |
| **NLR score** | 61,239 | 600 | 851,646 |  | 1.48(1.34,1.62) | <0.001 |  | 1.22(1.11,1.34) | <0.001 |
| **LMR score** | 180,315 | 1,513 | 2,506,818 |  | 1.46(1.34,1.59) | <0.001 |  | 1.06(0.97,1.16) | 0.180 |

**Abbreviations:** HR = hazard ratio; CI, confidence interval; NPS = Naples Prognostic Score; NLR=neutrophil-to-lymphocyte ratio; LMR=lymphocyte-to-monocyte ratio.

Crude model was unadjusted model.

Model 2 was adjusted for age, gender, white, education, townsend deprivation index group, drinking status, smoking status, body mass index group, physical activity, hypertension, diabetes, stroke, statin use,and polygenic risk score.

^a^All components were coded as dichotomous variables, with 0 designated as the reference group.

**Table S8.** Risk of incident Parkinson Disease according to genetic risk

| **Characteristics** | **Crude model** | |  | **Model 1** | |  | **Model 2** | |
| --- | --- | --- | --- | --- | --- | --- | --- | --- |
|  | **HR(95% CI)** | ***p* Value** |  | **HR(95% CI)** | ***p* Value** |  | **HR(95% CI)** | ***p* Value** |
| **Genetic risk**^a^ |  |  |  |  |  |  |  |  |
| Low | 1(Reference) |  |  | 1(Reference) |  |  | 1(Reference) |  |
| Intermediate | 1.34(1.20,1.50) | <0.001 |  | 1.34(1.20,1.50) | <0.001 |  | 1.34(1.20,1.50) | <0.001 |
| High | 1.91(1.72,2.12) | <0.001 |  | 1.91(1.72,2.12) | <0.001 |  | 1.91(1.72,2.12) | <0.001 |
| **P for trend** |  | <0.001 |  |  | <0.001 |  |  | <0.001 |
| **Per SD increase** | 1.37(1.32,1.43) | <0.001 |  | 1.37(1.32,1.43) | <0.001 |  | 1.38(1.32,1.43) | <0.001 |

Abbreviations: PD, Parkinson’s disease; HR, hazard ratio; CI, confidence interval; SD, standard deviation.

Crude model was unadjusted model.

Model 1 was adjusted for age, gender, white.

Model 2 was adjusted for Model 1 plus education, townsend deprivation index group, drinking status, smoking status, body mass index group, physical activity, hypertension, stroke,diabetes, and statin use.

^a^The genetic risk of PD was categorized as low, intermediate, or high based on tertiles of the polygenic risk score.

**Table S9.** Association Between NPS and Incident Parkinson Disease Using Inverse Probability Weighting (IPW) in the UK Biobank

| **Characteristics** | **Crude model** | |  | **Model 1** | |  | **Model 2** | |
| --- | --- | --- | --- | --- | --- | --- | --- | --- |
|  | **HR(95% CI)** | ***p* Value** |  | **HR(95% CI)** | ***p* Value** |  | **HR(95% CI)** | ***p* Value** |
| **Per 1-point increase^a^** | 1.37(1.31, 1.43) | <0.001 |  | 1.14 (1.09, 1.20) | <0.001 |  | 1.11 (1.06,1.17) | <0.001 |
| **NPS** |  |  |  |  |  |  |  |  |
| 0 | 1(reference) |  |  | 1(reference) |  |  | 1(reference) |  |
| 1 | 1.30(1.17, 1.43) | <0.001 |  | 1.05 (0.95, 1.17) | 0.309 |  | 1.04 (0.94, 1.15) | 0.439 |
| 2 | 1.74(1.57, 1.94) | <0.001 |  | 1.19 (1.06, 1.33) | 0.003 |  | 1.14 (1.01, 1.28) | 0.030 |
| 3 | 2.79(2.40, 3.24) | <0.001 |  | 1.62 (1.38, 1.90) | <0.001 |  | 1.47 (1.25, 1.74) | <0.001 |
| 4 | 3.57(2.11, 6.07) | <0.001 |  | 1.89 (1.11, 3.22) | 0.0019 |  | 1.71 (1.00, 2.92) | 0.052 |
| P for trend |  | <0.001 |  |  | <0.001 |  |  | <0.001 |

**Abbreviations:** HR = hazard ratio; CI, confidence interval; NPS = Naples Prognostic Score.

Crude model was unadjusted model.

Model 1 was adjusted for age, gender, white.

Model 2 was adjusted for Model 1 plus education, townsend deprivation index group, drinking status, smoking status, body mass index group, physical activity, hypertension, diabetes, stroke, statin use, and polygenic risk score.

^a^ Per 1-point increment in the NPS (0–4 points).

**Table S10.** Association Between NPS and Incident Parkinson Disease After Multiple Imputation for Missing Covariates in the UK Biobank

| **Characteristics** | **Crude model** | |  | **Model 1** | |  | **Model 2** | |
| --- | --- | --- | --- | --- | --- | --- | --- | --- |
|  | **HR(95% CI)** | ***p* Value** |  | **HR(95% CI)** | ***p* Value** |  | **HR(95% CI)** | ***p* Value** |
| **Per 1-point increase^a^** | 1.37(1.32, 1.42) | <0.001 |  | 1.15 (1.10, 1.20) | <0.001 |  | 1.11 (1.06,1.15) | <0.001 |
| **NPS** |  |  |  |  |  |  |  |  |
| 0 | 1(reference) |  |  | 1(reference) |  |  | 1(reference) |  |
| 1 | 1.31(1.19, 1.43) | <0.001 |  | 1.07 (0.98, 1.17) | 0.150 |  | 1.05 (0.96, 1.15) | 0.287 |
| 2 | 1.76(1.60, 1.94) | <0.001 |  | 1.21(1.10, 1.34) | <0.001 |  | 1.15 (1.04, 1.27) | 0.008 |
| 3 | 2.74(2.39, 3.14) | <0.001 |  | 1.61 (1.39, 1.85) | <0.001 |  | 1.42 (1.22, 1.65) | <0.001 |
| 4 | 3.56(2.23, 5.68) | <0.001 |  | 1.95 (1.22, 3.11) | 0.005 |  | 1.73 (1.08, 2.77) | 0.022 |
| P for trend |  | <0.001 |  |  | <0.001 |  |  | <0.001 |

**Abbreviations:** HR = hazard ratio; CI, confidence interval; NPS = Naples Prognostic Score.

Crude model was unadjusted model.

Model 1 was adjusted for age, gender, white.

Model 2 was adjusted for Model 1 plus education, townsend deprivation index group, drinking status, smoking status, body mass index group, physical activity, hypertension, diabetes, stroke, statin use, and polygenic risk score.

^a^ Per 1-point increment in the NPS (0–4 points).

**Table S11.** Risk of Incident Parkinson Disease According to NPS and Levels, Excluding PD Cases Occurring in the First 2 Years of Follow-up(n=316,985)

| **Characteristics** | **Crude model** | |  | **Model 1** | |  | **Model 2** | |
| --- | --- | --- | --- | --- | --- | --- | --- | --- |
|  | **HR(95% CI)** | ***p* Value** |  | **HR(95% CI)** | ***p* Value** |  | **HR(95% CI)** | ***p* Value** |
| **Per 1-point increase^a^** | 1.39(1.33,1.46) | <0.001 |  | 1.16(1.11,1.22) | <0.001 |  | 1.13(1.07,1.18) | <0.001 |
| **NPS** |  |  |  |  |  |  |  |  |
| 0 | 1(Reference) |  |  | 1(Reference) |  |  | 1(Reference) |  |
| 1 | 1.28(1.15,1.42) | <0.001 |  | 1.05(0.94,1.17) | 0.410 |  | 1.03(0.93,1.15) | 0.560 |
| 2 | 1.76(1.57,1.97) | <0.001 |  | 1.21(1.07,1.36) | 0.002 |  | 1.16(1.02,1.31) | 0.020 |
| 3 | 2.91(2.48,3.42) | <0.001 |  | 1.7(1.43,2.01) | <0.001 |  | 1.53(1.28,1.82) | <0.001 |
| 4 | 4.43(2.58,7.59) | <0.001 |  | 2.34(1.36,4.01) | 0.002 |  | 2.14(1.24,3.67) | 0.010 |
| P for trend |  | <0.001 |  |  | <0.001 |  |  | <0.001 |

**Abbreviations:** HR = hazard ratio; CI, confidence interval; NPS = Naples Prognostic Score.

Crude model was unadjusted model.

Model 1 was adjusted for age, gender, white.

Model 2 was adjusted for Model 1 plus education, townsend deprivation index group, drinking status, smoking status, body mass index group, physical activity, hypertension, diabetes, stroke, statin use, and polygenic risk score.

^a^ Per one-point increment in the NPS (0–4 points).

**Table S12.** Risk of Incident Parkinson Disease According to NPS and Levels, Excluding PD Cases Occurring in the First 5 Years of Follow-up(n=316,772)

| **Characteristics** | **Crude model** | |  | **Model 1** | |  | **Model 2** | |
| --- | --- | --- | --- | --- | --- | --- | --- | --- |
|  | **HR(95% CI)** | ***p* Value** |  | **HR(95% CI)** | ***p* Value** |  | **HR(95% CI)** | ***p* Value** |
| **Per 1-point increase^a^** | 1.41(1.34,1.48) | <0.001 |  | 1.18(1.12,1.24) | <0.001 |  | 1.14(1.08,1.20) | <0.001 |
| **NPS** |  |  |  |  |  |  |  |  |
| 0 | 1(Reference) |  |  | 1(Reference) |  |  | 1(Reference) |  |
| 1 | 1.27(1.14,1.42) | <0.001 |  | 1.04(0.93,1.17) | 0.510 |  | 1.02(0.91,1.15) | 0.690 |
| 2 | 1.79(1.59,2.02) | <0.001 |  | 1.23(1.08,1.40) | 0.001 |  | 1.17(1.03,1.33) | 0.020 |
| 3 | 2.97(2.51,3.52) | <0.001 |  | 1.73(1.45,2.07) | <0.001 |  | 1.55(1.29,1.86) | <0.001 |
| 4 | 5.13(3.02,8.70) | <0.001 |  | 2.71(1.59,4.60) | <0.001 |  | 2.26(1.30,3.94) | 0.004 |
| P for trend |  | <0.001 |  |  | <0.001 |  |  | <0.001 |

**Abbreviations:** HR = hazard ratio; CI, confidence interval; NPS = Naples Prognostic Score.

Crude model was unadjusted model.

Model 1 was adjusted for age, gender, white.

Model 2 was adjusted for Model 1 plus education, townsend deprivation index group, drinking status, smoking status, body mass index group, physical activity, hypertension, diabetes, stroke, statin use, and polygenic risk score.

^a^ Per one-point increment in the NPS (0–4 points).

**Table S13.** Risk of Incident Parkinson Disease According to NPS and Levels, Including Participants With Cancer at Baseline (n=345,078)

| **Characteristics** | **Crude model** | |  | **Model 1** | |  | **Model 2** | |
| --- | --- | --- | --- | --- | --- | --- | --- | --- |
|  | **HR(95% CI)** | ***p* Value** |  | **HR(95% CI)** | ***p* Value** |  | **HR(95% CI)** | ***p* Value** |
| **Per 1-point increase^a^** | 1.38(1.32,1.44) | <0.001 |  | 1.15(1.10,1.20) | <0.001 |  | 1.11(1.06,1.17) | <0.001 |
| **NPS** |  |  |  |  |  |  |  |  |
| 0 | 1(Reference) |  |  | 1(Reference) |  |  | 1(Reference) |  |
| 1 | 1.29(1.17,1.43) | <0.001 |  | 1.06(0.95,1.17) | 0.300 |  | 1.04(0.94,1.16) | 0.420 |
| 2 | 1.74(1.56,1.94) | <0.001 |  | 1.19(1.06,1.33) | 0.003 |  | 1.14(1.01,1.28) | 0.030 |
| 3 | 2.83(2.43,3.30) | <0.001 |  | 1.64(1.40,1.92) | <0.001 |  | 1.49(1.26,1.75) | <0.001 |
| 4 | 3.67(2.17,6.22) | <0.001 |  | 1.93(1.14,3.27) | 0.010 |  | 1.77(1.04,3.00) | 0.040 |
| P for trend |  | <0.001 |  |  | <0.001 |  |  | <0.001 |

**Abbreviations:** HR = hazard ratio; CI, confidence interval; NPS = Naples Prognostic Score.

Crude model was unadjusted model.

Model 1 was adjusted for age, gender, white.

Model 2 was adjusted for Model 1 plus education, townsend deprivation index group, drinking status, smoking status, body mass index group, physical activity, hypertension, diabetes, cancer, stroke, statin use, and polygenic risk score.

^a^ Per one-point increment in the NPS (0–4 points).

**Table S14.** Risk of incident PD according to NPS and levels with stratifying the follow-up period into two intervals: 0-5 years and >5 years

| **Characteristics** | **Follow-up intervals: 0-5 years**  **(n = 4,212; cases = 235)** | |  | **Follow-up intervals > 5 years**  **(n = 312,820; cases = 2,012)** | |
| --- | --- | --- | --- | --- | --- |
|  | **HR(95% CI)** | ***p* Value** |  | **HR(95% CI)** | ***p* Value** |
| **Per 1-point increase^a^** | 1.13(0.96,1.34) | 0.130 |  | 1.14(1.08,1.20) | <0.001 |
| **NPS** |  |  |  |  |  |
| 0 | 1(Reference) |  |  | 1(Reference) |  |
| 1 | 1.49(1.06,2.09) | 0.020 |  | 1.02(0.91,1.15) | 0.690 |
| 2 | 1.35(0.91,2.01) | 0.130 |  | 1.17(1.03,1.33) | 0.020 |
| 3 | 1.54(0.85,2.78) | 0.150 |  | 1.55(1.29,1.86) | <0.001 |
| 4 | Not estimable (insufficient events) | - |  | 2.26(1.30,3.94) | 0.004 |
| P for trend |  | 0.130 |  |  | <0.001 |

**Abbreviations:** HR = hazard ratio; CI, confidence interval; NPS = Naples Prognostic Score.

Adjusted for age, gender, white, education, townsend deprivation index group, drinking status, smoking status, body mass index group, physical activity, hypertension, diabetes, stroke, statin use, and polygenic risk score.

^a^ Per one-point increment in the NPS (0–4 points).
